# Voluntary Adolescent-Onset Alcohol Drinking Fails to Influence Alcohol Consumption or Anxiety-Like Behaviour in Adulthood in Female Alcohol-Preferring Rats

**DOI:** 10.1093/alcalc/agab063

**Published:** 2021-08-31

**Authors:** Ekaterina Mugantseva, Petri Hyytiä, Antti Latvala

**Affiliations:** Institute for Molecular Medicine Finland (FIMM), University of Helsinki, P.O. Box 20 (Tukholmankatu 8), FI-00014 Helsinki, Finland; Institute of Theoretical and Experimental Biophysics RAS, Institutskaya, 3, Pushchino, 142290, Moscow region, Russia; Department of Pharmacology, Medicum, University of Helsinki, P.O. Box 63 (Haartmaninkatu 8), FI-00014 Helsinki, Finland; Institute for Molecular Medicine Finland (FIMM), University of Helsinki, P.O. Box 20 (Tukholmankatu 8), FI-00014 Helsinki, Finland; Institute of Criminology and Legal Policy, University of Helsinki, P.O. Box 16 (Snellmaninkatu 10), FI-00014 Helsinki, Finland

## Abstract

**Aims:**

Alcohol exposure during adolescence is associated with both increased risk for alcohol use disorders and anxiety in adulthood. Our present experiments examined this association using alcohol-preferring AA (Alko Alcohol) rats selected for high voluntary alcohol drinking.

**Methods:**

Two groups of female AA rats acquired alcohol drinking at different ages. We gave the adolescent-onset group free choice to 10% alcohol and water for seven weeks, starting on post-natal day 42 (PND 42), whereas the adult-onset group started drinking alcohol on PND 112. After the 7-week drinking, we withdrew the adolescent group from alcohol for two weeks, followed by another voluntary 7-week drinking period, started at the same age as the adult-onset group. We assessed anxiety-like behaviour repeatedly during alcohol drinking with open field and elevated plus maze tests. At the end of alcohol drinking, we also tested the rats using the light/dark box, stress-induced body temperature test and social dominance test.

**Results:**

During the first 7-week alcohol drinking, adolescent rats exhibited significantly slower acquisition of alcohol drinking and lower alcohol preference than the adult-onset group. However, when tested at the same age as the adult-onset rats, they displayed identical alcohol intake and preference. We found no alcohol-induced effects on anxiety- or stress-related behaviour in the experimental groups at any time points.

**Conclusions:**

These data show that the genetically determined phenotype of high alcohol drinking of the female alcohol-preferring AA rats is not associated with a predisposition to develop anxiety-like behaviour following voluntary alcohol exposure, even when initiated during adolescence.

## Teaser

**Short Summary:** We tested the effects of adolescent-onset alcohol drinking on the level of alcohol intake and anxiety-like behaviour in adulthood in female alcohol-preferring rats. Compared with adult-onset rats, adolescent rats exhibited an identical trajectory of alcohol consumption. In addition, early alcohol exposure did not influence anxiety-like behaviours in adulthood.

## INTRODUCTION

Adolescence is a critical period for brain development and maturation. In both humans and experimental animals, adolescent brains are particularly sensitive to the effects of binge-type alcohol drinking that leads to exacerbated brain damage (Crews et al. 2000) and learning problems (Sircar and Sircar 2005). Epidemiological studies show that early initiation of alcohol use increases the future risk of developing alcohol use disorders (DeWit et al. 2000; Dawson et al. 2008). This relationship between adolescent and adult alcohol consumption has been found also in animal models (Towner and Varlinskaya 2020).

Alcohol use disorders and anxiety disorders exhibit remarkable co-occurrence (Vorspan et al. 2015). Epidemiological studies have been unable to unravel the causes of this comorbidity (Lai et al. 2015), but studies utilizing genetically informed research designs have suggested that it is not fully explained by shared genetic risks between anxiety and alcohol use disorders (Virtanen et al. 2020). The association of adolescent alcohol use and increased prevalence of anxiety in adulthood suggests that anxiety could be a consequence of repeated alcohol intoxication and withdrawal during the vulnerable period of brain maturation. Nevertheless, alcohol has anxiolytic properties, and therefore, using alcohol for alleviating distress could increase the risk for alcohol use disorders (Kushner et al. 2000).

In the present study, we aimed at investigating the effects of long-term adolescent alcohol exposure on subsequent alcohol drinking and anxiety-like behaviour. Instead of experimenter-administered alcohol, we employed a model in which rats could voluntarily consume high amounts of alcohol for long periods. This can be achieved by a genetically selected rat line, such as alcohol-preferring AA rats developed by selective breeding (Sommer et al. 2006). AA rats quickly learn to drink pharmacologically relevant amounts of alcohol (5–8 g/kg/day), leading to blood alcohol levels as high as 50 mg% that induce psychomotor stimulation (Päivärinta and Korpi 1993; Nurmi et al. 1999). Several neurochemical and behavioural traits have co-segregated with alcohol preference in this line, including impulsivity and risk-taking (Möller et al. 1997; Roman et al. 2012). These behavioural traits separate them from such selected lines as the Sardinian preferring line (sP) and its substrain, the Marchigian-Sardinian alcohol-preferring rats (msP) that exhibit an innate anxiogenic- and depression-like behaviour (Colombo et al. 1995; Ciccocioppo et al. 2006), which is attenuated by alcohol consumption (Colombo et al. 1995). However, the effects of long-term alcohol use on anxiety or depressive behaviours in AA rats are not known.

Alcohol consumption is known to have differential effects on men and women. Women exhibit greater negative emotions such as anxiety and depression related to alcohol drinking (Rubonis et al. 1994), and the association between alcohol use disorders and anxiety disorders is stronger in women than in men (Virtanen et al. 2020). Rodent studies showing depressive- and anxiety-like behaviours after prolonged alcohol exposure have mostly been conducted using male animals. Given that female rodents generally exhibit higher alcohol intake relative to body weight (Lancaster and Spiegel 1992; Almeida et al. 1998) and display more pronounced withdrawal-related experimental anxiety (Li et al. 2019), more work is needed to describe the development of anxiety-related behaviours across chronic alcohol drinking in females. Consequently, we performed the present studies using female AA rats. Collectively, we aimed at clarifying the relationship between alcohol and anxiety-like behaviour longitudinally across weeks of alcohol drinking and the interaction of alcohol exposure with the age of onset of drinking, concentrating specifically on female rats.

## MATERIALS AND METHODS

### Animals

We used a total of 48 female alcohol-preferring AA (Alko Alcohol) rats bred at the University of Helsinki. Rats were housed in a room with a controlled temperature of 21 ± 1°C and a relative humidity of 55 ± 10% on a 12-h light/dark cycle with lights on at 06:00 h. Rats had ad libitum access to pellet food and tap water except for the first 4 days during the initiation of alcohol drinking. Rats were housed individually and received at least a week of acclimatization and handling before the experiment. All experimental procedures using animals were conducted in accordance with directive 2010/63/EU of the European Parliament and of the Council and the Finnish Act on the Protection of Animals Used for Science or Educational Purposes (497/2013) and were approved by the project authorization board of the Regional State Administration Agency for Southern Finland.

### Study design

**Fig. 1:**
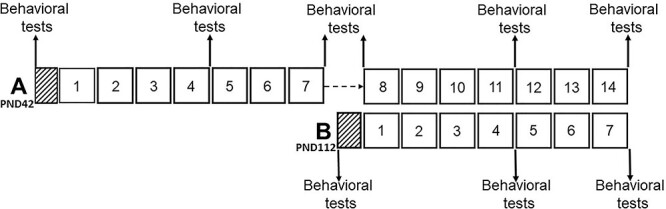
The design of the present experiments; the diagram depicts the adolescent-onset group A and the adult-onset group B; the numbered squares indicate the weeks of voluntary free-choice alcohol drinking and the hatched squares the 4-day forced alcohol drinking phase; arrows mark the times of the behavioural tests.

We divided the 48 rats randomly into two groups of 24 animals (the adolescent-onset and adult-onset groups) and then further divided these groups into the control and alcohol-drinking groups, each consisting of 12 subjects. For the adolescent-onset rats, we started the alcohol-drinking procedure at the age of 6 weeks, i.e. at postnatal day 42 (PND42), whereas the adult-onset group started the experiment at the age of 16 weeks, PND112 (see the experimental design depicted in Fig. 1). At the beginning of the experiments, the adolescent-onset rats weighed 120 ± 6 g, whereas the adult-onset rats weighed 210 ± 13 g. Both adolescent- and adult-onset groups could drink either alcohol or water for seven weeks (Fig. 1). After this period, the adolescent-onset group had a break for two weeks, after which alcohol drinking was resumed. This period lasted also for seven weeks, and it was started at the same age as the adult-onset alcohol exposure (Fig. 1). Both the adolescent-onset and adult-onset groups were tested using behavioural tests (see below) three times during the 7-week alcohol exposures: before the alcohol exposure (baseline) and both following four and seven weeks of alcohol exposure. In addition, the adolescent-onset rats were retested both at the beginning, in the middle and the end of the second 7-week drinking period. Behavioural tests were conducted during the light period, at least 4 hours after the end of the dark period. In all testing, we mixed water- and alcohol-exposed groups to minimize environmental influence and always conducted the least stressful tests first. Because alcohol drinking by the AA rats is largely limited to the dark period and alcohol access was denied during the 1-h habituation prior to behavioural tests, we assumed that the subjects had no pharmacologically relevant blood alcohol levels during testing.

### Alcohol drinking procedure

The drinking fluids were provided in 250-mL drinking bottles equipped with stainless steel spouts and located in the food tray of the cage covers. Rats of the alcohol groups (*n* = 24) were given 10% (v/v) ethyl alcohol as their only drinking fluid during the first 4 days, which is suggested to promote learning of the reinforcing properties of alcohol and habituation to alcohol taste (Eriksson 1968). After this phase, rats were allowed a two-bottle choice between alcohol and water for the following 7 weeks. Once per week, we recorded the consumption of the fluids and body weights, refilled the bottles with fresh solutions and changed the left–right position of the bottles to avoid the development of side preference. During this phase, rats in the control groups (*n* = 24) had access to water from the two drinking bottles.

### Behavioural tests

#### Open field test

The open field consisted of a circular arena, 85 cm in diameter and with a 50-cm high wall. After a 1-h habituation in the room, animals were placed individually in the centre of the arena and observed for 10 min. The Ethovision® XT video tracking system (Version 4.0, Noldus Information Technology, The Netherlands) was used for recording the total distance travelled and the time spent in the centre zone of the field (a circular area with a diameter of 51 cm). After each individual experimental session, the arena was wiped with a 2% hydrogen peroxide solution to get rid of any smell left by the animal.

#### Elevated plus maze test

The apparatus used in the present study was made of acrylic and consisted of a central square (10 × 10 cm) and four perpendicular arms (10 × 50 cm each), with two of them with 40-cm-high walls shielding the arms (closed arms), and two of them without walls (open arms). The maze was elevated to a height of 60 cm from the floor. We placed each animal in the centre of the maze with its head facing one of the open arms and, upon release, allowed it to move freely throughout the maze for 5 min, while its behaviour was video recorded. The apparatus was cleaned with 2% hydrogen peroxide before introducing each animal. The EthoVision software was used for recording behaviour and extracting data from the sessions.

#### Light/dark box test

The light/dark box test was carried out in an experimental arena consisting of two compartments (each 26.4 × 20.6 cm), one of which was dark and the other illuminated. Dark and light compartments were connected by a dark centre chamber (15.9 × 20.6 cm) allowing free movement between the compartments. Rats were individually released into the light compartment and allowed to explore freely all compartments for 10 min. The time spent in each compartment during the test was recorded using a computer-controlled animal movement tracking device using three 16-beam infrared arrays (Med Associates, St. Albans, VA, USA). All tests measuring anxiety-related behaviour were conducted under dim indirect light during the light phase of the 12-h light/dark cycle.

#### Social dominance tube test

We used a transparent plastic 60-cm-long tube with the inner diameter of 7 cm, which is sufficient to permit an adult rat to pass through the tube without reversing direction. During the test, two rats were released simultaneously into the opposite ends of the tube. In most cases, one rat would exhibit dominance and force the other to withdraw from the tube. The rat that caused the opponent to withdraw within 2 min was designated the ‘winner’. In rare cases, when neither rat left the tube within 2 min, the test ended in a draw. The percentage of retreats and forwards was calculated from the total number of encounters. Twelve alcohol rats and twelve control rats were used in each experiment. Each animal was tested against 11 animals of the other group from different cages. Between each trial, the tube was cleaned.

#### Stress induced hyperthermia test

We carried out body temperature recordings in the stable thermal environment of the testing room. Rectal temperature was measured by gently inserting a digital thermistor probe (CMA/150 Temperature Controller: CMA/Microdialysis, Solna, Sweden) to intrarectal until a stable reading was obtained. After the baseline measurement, we restrained the animals in Plexiglas rodent restrainers (Stoelting, Wood Dale, IL) for 30 minutes and measured body temperature following 15 and 30 min of restraint. The restrainers had holes in the walls to allow heat dissipation. The back wall of the restrainer was movable to accommodate animals of different sizes and to keep them immobilized, with the tail extending beyond the back wall. For each individual rat, the baseline temperature (T1), the 30-min restraint temperature (T2) and their difference (ΔT) = T2 − T1 were determined.

### Statistics

Behavioural data were analysed with three-way analyses of variance (ANOVA), with the age of onset (adolescent, adult) and exposure (control, alcohol) as between-subjects factors and time as the within-subjects factor. For comparing two groups across repeated tests, two-way (group, time) repeated measures ANOVAs were used and for comparison of two groups at a single time point, independent *t*-tests. In addition, one-way ANOVAs were used for describing performance by single experimental groups across time points. Results were considered statistically significant at *P* < 0.05.

## RESULTS

**Fig. 2:**
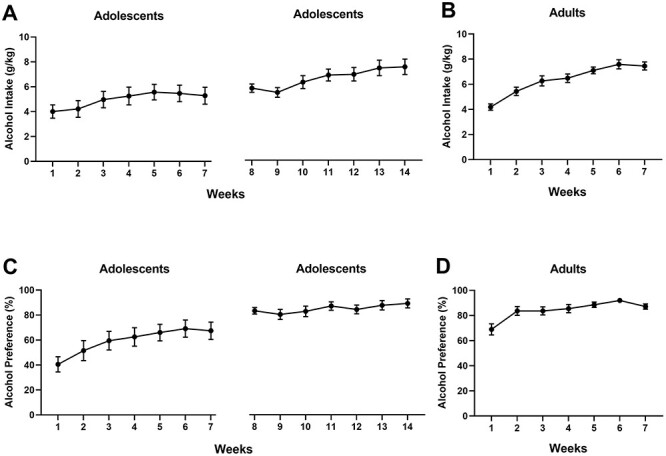
Acquisition of alcohol drinking by adolescent and adult female AA rats; (A) alcohol consumption by adolescent rats during the two 7-week alcohol access periods, and (B) alcohol consumption by adult rats; (C) alcohol preference of adolescent rats during the alcohol access weeks, and (D) alcohol preference by the adult rats; all data are depicted as mean intake or preference ± SEM.

During the first week of alcohol access, the adolescent and adult rats exhibited virtually identical alcohol consumption (adolescent rats 4.01 ± 0.54 g/kg/day, adult rats 4.19 ± 0.25 g/kg/d, mean ± SEM) (Fig. 2A and B). Both adolescent (*F*_6,66_ = 5.30, *P* < 0.0001) and adult rats (*F*_6,66_ = 31.71, *P* < 0.0001) increased their consumption across the first seven alcohol access weeks. However, comparison of age groups across these weeks revealed that the adult rats increased their alcohol drinking more rapidly, as indicated by a significant main effect of age (*F*_1,22_ = 4.41, *P* = 0.045) and a significant time X age interaction (*F*_6,132_ = 3.82, *P* = 0.002). Likewise, Fig. 2C and D show that both adolescent (*F*_6,66_ = 15.34, *P* < 0.0001) and adult rats (*F*_6,66_ = 10.44, *P* < 0.0001) increased their alcohol preference over the initial weeks. Moreover, the adult rats exhibited a higher preference (*F*_1,22_ = 12.37, *P* = 0.002) that developed in parallel with that of the adolescent rats (time × age interaction, *F*_6,132_ = 1.45, *P* = 0.20). In adult rats, elevated alcohol drinking was accompanied by increased total fluid intake compared with the control rats (*F*_1,22_ = 22.81, *P* < 0.0001) (data not shown), whereas the adolescent groups drank identical amounts of fluid (*F*_1,22_ = 1.62, *P* = 0.22). Overall, the younger rats had a higher total fluid intake over the experimental weeks (*F*_1,44_ = 37.76, *P* < 0.0001).

After a 2-week break, adolescent rats resumed alcohol drinking at the age of 16 weeks, which was the age when the adult-onset group had first been given alcohol access. Therefore, we were able to compare adolescent-onset subjects with age-matched adult-onset subjects (drinking weeks 8–14 in panel A vs. weeks 1–7 in panel B). During these additional seven weeks, the adolescent rats further increased both their alcohol intake (*F*_6,60_ = 13.13, *P* < 0.0001) and preference (*F*_6,60_ = 3.87, *P* = 0.002). However, opposite to the initial seven weeks, now the age groups exhibited no differences in alcohol drinking (*F*_1,21_ = 0.05, *P* = 0.82) or alcohol preference (*F*_1,21_ = 0.002, *P* = 0.97). A significant age × time interaction for alcohol intake (*F*_6,126_ = 3.76, *P* = 0.002) revealed an initial increase in the adolescent-onset group during the first access week after the break. This temporary increase during the first week was probably produced by denying the rats access to alcohol, a phenomenon known as the alcohol deprivation effect.

**Fig. 3:**
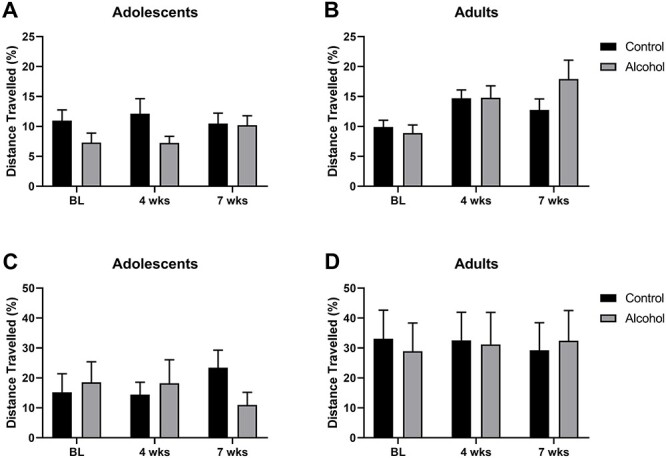
Open-field and elevated plus-maze behaviour in adolescent and adult female AA rats; measurements were made prior to the 7-week exposure to voluntary alcohol, during alcohol drinking (week 4), and immediately after it at the end of week 7; shown are open field centre distance travelled by adolescent (A) and adult (B) rats, and plus maze open arm distance travelled by adolescent (C) and adult (D) rats; both open-field centre distance and plus-maze open arm distance were expressed as percentage of the global locomotor activity during each behavioural test; the data bars denote means ± SEM.

To evaluate the effects of voluntary alcohol drinking on anxiety-like behaviour, we conducted a battery of anxiety tests repeatedly during the alcohol drinking weeks, i.e. at baseline, at week 4 and at the end of the drinking period at week 7. Because rats of all ages exhibited decreased locomotor activity (adolescent rats: *F*_2,44_ = 40.31, *P* < 0.0001; adult rats: *F*_2,44_ = 28.58, *P* < 0.0001) in the open-field arena across time, we expressed centre zone behaviour as a percentage of distance travelled of the total distance in the arena (for non-normalized data, see Supplemental Figure 1A and B). Figure 3A and B shows that across the three time points (baseline, 4 weeks, 7 weeks), both adolescent and adult rats increased their exploration in the central zone (*F*_2,88_ = 4.96, *P* = 0.009), but the age groups exhibited parallel increases (*F*_2,88_ = 2.58, *P* = 0.82) and showed no effect from the alcohol exposure (*F*_1,44_ = 0.48, *P* = 0.49). However, adult rats spent significantly more time in the centre than the adolescent rats (*F*_1,44_ = 9.53, *P* = 0.003) across the measurements.

Similar to the open field, all groups decreased their overall exploration in the plus maze across repeated testing (adolescent rats: *F*_2,44_ = 20.19, *P* < 0.0001; adult rats: *F*_2,44_ = 14.10, *P* < 0.0001), prompting us to express the open arm exploration as a percentage of the total distance travelled in the maze (for non-normalized data, see Supplemental Figure 1C and D). Across the three measurements, we saw no effect of alcohol exposure (*F*_1,44_ = 0.05, *P* = 0.82), no change in behaviour across repeated measurements (*F*_2,88_ = 0, *P* = 1), but higher open arm activity by the adult than the adolescent rats (*F*_1,44_ = 6.51, *P* = 0.014) (Fig. 3C and D). In adolescent-onset rats, open-field tests conducted during the second drinking period (weeks 8, 11, 14) revealed no effect of alcohol exposure (*F*_1,21_ = 1.11, *P* = 0.30). Similarly, alcohol drinking failed to affect plus-maze behaviour during this period (*F*_1,22_ = 0.82, *P* = 0.38) (data not shown).

**Fig. 4:**
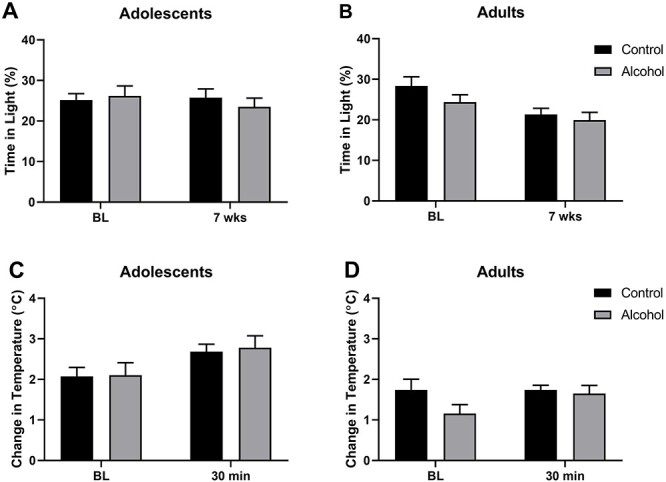
Light–dark box behaviour and immobilization stress-induced body temperature change in adolescent and adult female AA rats; measurements were made prior to and following the 7-week exposure to voluntary alcohol; shown are mean ± SEM percentage of time spend in the light compartment by (A) adolescent and (B) adult rats and mean ± SEM temperature change in adolescent (C) and (D) adult rats.

The light/dark box tests were performed only twice, during the baseline before alcohol access and at the end of the 7-week drinking phase, whereas the restraint stress induced hyperthermia tests were conducted at the end of the drinking period. In the light/dark box, we saw no effect by the alcohol exposure (*F*_1,44_ = 1.41, *P* = 0.24), but a significant change across the two measurements (*F*_1,44_ = 5.63, *P* = 0.02) (Fig. 4 A and B). This was produced by significantly reduced time spent in the light compartment by the adult rats during the final 7-week test (*F*_1,22_ = 8.94, *P* = 0.007). Stress-induced hyperthermia tests shown in Fig. 4C and D did not show any effect of alcohol exposure either (*F*_1,44_ = 0.46, *P* = 0.51), but the adolescent groups exhibited a significant increase in body temperature at the 30-min time point (*F*_1,23_ = 20.30, *P* = 0.0002).

**Fig. 5:**
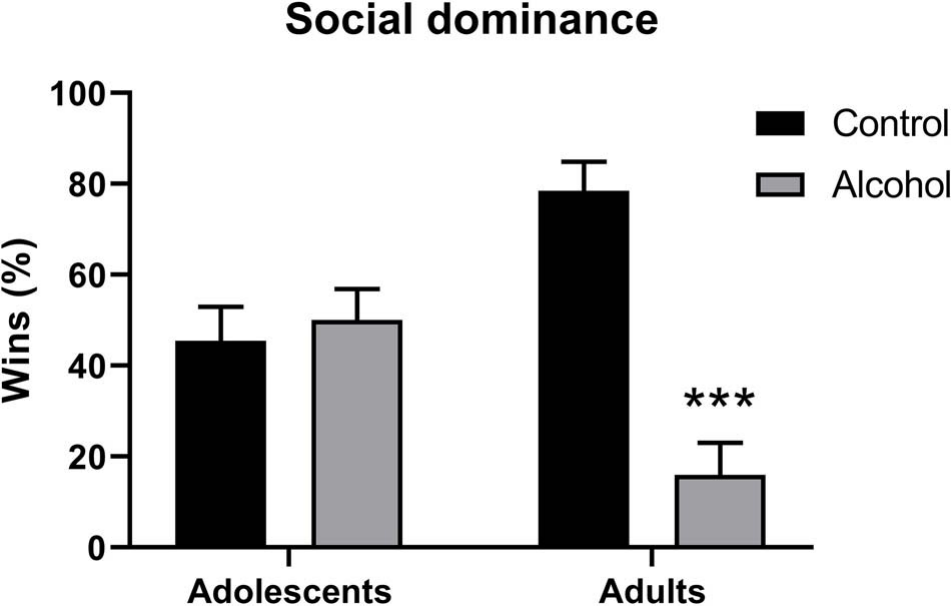
Social dominance measured using the tube test by female adolescent and adult rats following long-term voluntary water (control) or alcohol drinking at the end of the first 7-week alcohol access week; data are expressed as percentage ± SEM of wins (forward movement) during the test. ^*^^*^^*^P < 0.001, independent *t*-test.

We measured social dominance of the experimental groups using the tube test shown in Fig. 5. These data revealed no significant effect of age (*F*_1,43_ = 2.99, *P* = 0.94), but a significant effect of alcohol exposure (*F*_1,43_ = 17.29, *P* = 0.0002), as well as the age × exposure interaction (*F*_1,43_ = 23.13, *P* < 0.0001) that was produced by a significant dominance of the adult control rats compared with the alcohol-exposed subjects (*t*_22_ = 6.57, *P* < 0.0001). The dominance by the control rats could be due to their more rapid weight gain across the 7 weeks, indicated by a significant time × exposure interaction (*F*_6,132_ = 18.90, *P* = 0.038) for body weight. During the social dominance testing week, rats of the control group were slightly heavier than the alcohol group (*t*_22_ = 1.95, *P* = 0.065).

## DISCUSSION

We investigated the effect of the age of alcohol drinking onset on subsequent alcohol drinking and anxiety-like behaviour in female alcohol-preferring AA rats. We found that the adolescent-onset and adult-onset groups drank the same amount of alcohol during the first exposure week. During the following six weeks, however, the adolescent rats increased their alcohol drinking significantly less than the adult rats and exhibited significantly lower alcohol preference. In contrast, when we re-introduced the adolescent rats to alcohol again at the age at which the adult rats started their alcohol exposure, we found no difference between these age groups. Both in the adolescent and adult-onset groups, alcohol exposure had no effect on anxiety-like behaviour, measured by open field, plus maze and light/dark box tests conducted at various time points across weeks of alcohol exposure. In the adult-onset group, alcohol exposure appeared to render the rats socially submissive.

In humans, initiation of alcohol use in adolescence is associated with an elevated risk of developing alcohol use disorders, as well as depression and anxiety in adulthood (DeWit et al. 2000; Rohde et al. 2001; Dawson et al. 2008). The existing animal data on the relationship between early alcohol consumption and the subsequent behavioural alterations, including alcohol- and anxiety-related behaviours, remain conflicting and inconclusive. Important variables influencing the outcome of adolescent alcohol exposure appear to include the exposure method and the timing of alcohol exposure. Most studies, in which alcohol was administered using intragastric (IG) gavage or intraperitoneal (IP) injections during adolescence, reported increased alcohol drinking (Pascual et al. 2009; Maldonado-Devincci et al. 2010; Alaux-Cantin et al. 2013; Sakharkar et al. 2019) or anxiety-like behaviour during adulthood (Coleman Jr. et al. 2014; Pandey et al. 2015; Kokare et al. 2017; Loxton and Canales 2017; Torcaso et al. 2017; Kyzar et al. 2019). In contrast, as reviewed recently, approximately only a third of the studies using voluntary alcohol consumption models have been able to demonstrate increased alcohol intake when recorded later in adulthood (Towner and Varlinskaya 2020). For example, intermittent 20% free-choice alcohol drinking by adolescent Wistar rats increased their alcohol consumption later in life (Amodeo et al. 2017) and free-choice 15% alcohol drinking by adolescent female alcohol-preferring P rats exhibited increased acquisition of operant alcohol self-administration, suggesting enhanced alcohol reinforcement (Rodd-Henricks et al. 2002). With respect to anxiety-like behaviour, adolescent voluntary alcohol drinking models have generally not been successful in increasing anxiety when measured in adulthood.

The critical factor determining the efficacy of adolescent alcohol exposure to influence behaviour in adulthood could be the blood alcohol concentration (BAC) attained upon alcohol administration. Generally, intermittent drinking paradigm or forced alcohol administration (injections, gavage) leading to BACs in the range of 100–200 mg/dl appears more successful in causing long-term behavioural changes than BACs of ~20–40 mg/dl measured during continual free-choice alcohol drinking (Towner and Varlinskaya 2020). The subjects of the present study, alcohol-preferring AA rats, exhibit BACs of ~20–50 mg/dl during voluntary alcohol drinking bouts (Nurmi et al. 1999), and it could therefore be argued that the BACs typical of this phenotype are not expected to induce neuronal changes conducive to increased alcohol drinking or anxiety-like behaviour. On the other hand, we aimed at examining the malleability of this phenotype using procedures based only on voluntary behaviour of these subjects. Thus, we can conclude that the failure to influence the acquisition of alcohol drinking by early alcohol exposure suggests that the acquisition curve is largely determined by genetic factors that are probably related to the learning of the reinforcing properties of alcohol. In the same vein, high BACs may also be critical for increasing adult anxiety-like behaviours following adolescent alcohol exposure. However, earlier behavioural characterization of naive AA rats suggests that alcohol preference in this rat line is associated with increased risk-taking behaviour, impulsivity and low anxiety that separates them from most alcohol-preferring rat lines produced by genetic selection (Roman et al. 2012). It is therefore possible that these rats are innately resistant to manipulations that seek to increase their anxiety levels. Furthermore, alcohol preference in this rat line does not appear to be genetically associated with high anxiety, unlike in Sardinian preferring sP or alcohol-preferring P rats (Stewart et al. 1993; Colombo et al. 1995).

Repeated tests for anxiety-like behaviour can be seen as a potential confounding factor in our experimental design. Rats exhibited diminished locomotion across repeated testing in the open field and elevated plus maze, reflecting either reduced environmental novelty or age-related alterations in activity levels, but these changes should not affect the ability to detect anxiety-like behaviour. Indeed, as observed in the test validation, rats can be tested at least three times in the elevated plus maze without significant loss of the unconditioned aversiveness of the test (Pellow et al. 1985). In addition, our tests were separated by at least three weeks, both the control and alcohol groups displayed identical habituation to the test and the light/dark box test with fewer testing sessions produced the same outcome.

Vulnerability of rodents to adolescent alcohol exposure has been suggested to depend on the timing of the exposure. In female rats, puberty occurs approximately from 36 to 39 days of age (Parker Jr. and Mahesh 1976), and thus, the period for early alcohol exposure during postnatal days 28–37 can be defined as peripubertal, whereas later time points correspond to mid and late puberty. There is evidence that alcohol exposure during the peripubertal period could have a greater effect on alcohol intake in adulthood than a later exposure (Ho et al. 1989; Alaux-Cantin et al. 2013; Fernandez et al. 2016). However, other reports have concluded that previous alcohol administration at any age promotes alcohol consumption measured later (Hefner and Holmes 2007; Carrara-Nascimento et al. 2014; Amodeo et al. 2017). Therefore, even if our alcohol exposure started during the mid and late puberty, we cannot conclude that the timing prevented us from seeing increases in adulthood. Sex differences have also been suggested to influence the vulnerability, with females being more susceptible to alcohol exposure effects than males, but few studies have directly compared females and males, and they do not unequivocally demonstrate greater female vulnerability to early alcohol effects (Maldonado-Devincci et al. 2010; Amodeo et al. 2018).

To conclude, in female alcohol-preferring AA rats selected for high voluntary alcohol consumption, initiation of alcohol drinking during mid-late puberty did not influence either their alcohol consumption or anxiety-like behaviour in adulthood, measured at multiple time points both during adolescence and adulthood. Similarly, alcohol drinking had no effects on anxiety in adult rats. Although the specific genetic make-up produced by genetic selection for high alcohol intake in these rats is not associated with predisposition to develop anxiety-like psychopathology, it is possible that other behavioural traits characteristic of these rats, such as risk taking and impulsivity, could be altered during long-term alcohol drinking.

## Supplementary Material

Supplemental_Material_agab063

## Data Availability

The data underlying this article will be shared on reasonable request to the corresponding author.
