# Supplementary material for: Voluntary Adolescent-Onset Alcohol Drinking Fails to Influence Alcohol Consumption or Anxiety-Like Behaviour in Adulthood in Female Alcohol-Preferring Rats
Source: Alcohol Alcohol. 2021 Aug 31;57(3):396–403. doi: 10.1093/alcalc/agab063 (PMC9086760; doi:10.1093/alcalc/agab063)
Supplement: Supplemental_Material_agab063 [file supplemental_material_agab063.docx]

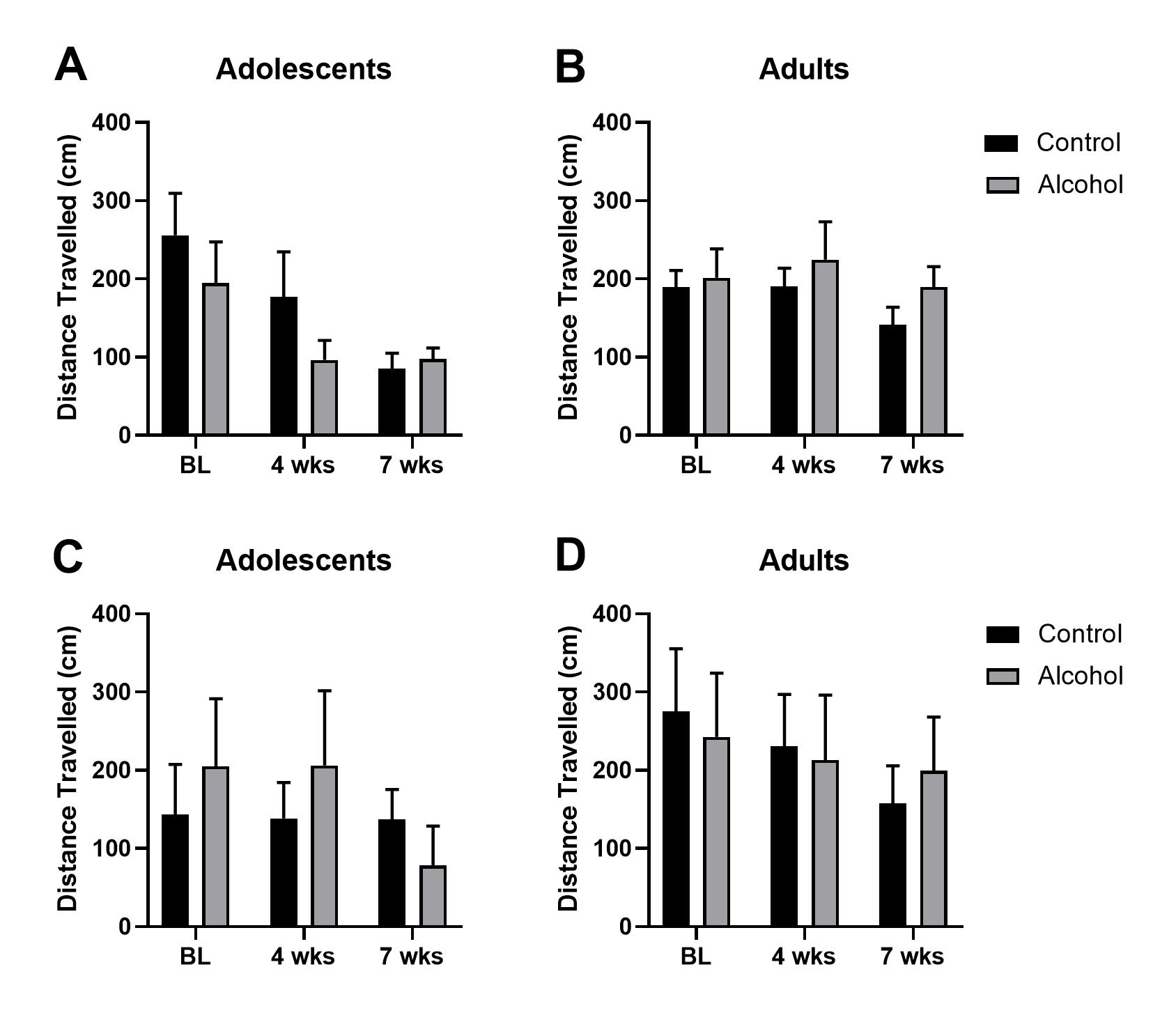


**Supplemental Figure 1.** Open-field and elevated plus-maze behaviour in adolescent and adult female AA rats. Measurements were made prior to the 7-week exposure to voluntary alcohol, during alcohol drinking (week 4), and immediately after it at the end of week 7. Shown are open field centre distance travelled by adolescent (A) and adult (B) rats, and plus maze open arm distance travelled by adolescent (C) and adult (D) rats. The data bars denote means ± SEM.
